# Metformin and Berberine Prevent Olanzapine-Induced Weight Gain in Rats

**DOI:** 10.1371/journal.pone.0093310

**Published:** 2014-03-25

**Authors:** Yueshan Hu, Alan J. Young, Erik A. Ehli, Dustin Nowotny, Paige S. Davies, Elizabeth A. Droke, Timothy J. Soundy, Gareth E. Davies

**Affiliations:** 1 Avera Institute for Human Genetics, Avera McKennan Hospital & University Health Center, Sioux Falls, South Dakota, United States of America; 2 Department of Psychiatry, Sanford School of Medicine, University of South Dakota, Sioux Falls, South Dakota, United States of America; 3 Department of Veterinary and Biomedical Sciences, College of Agriculture & Biological Sciences, South Dakota State University, Brookings, South Dakota, United States of America; 4 Department of Health and Nutritional Sciences, College of Education & Human Sciences, South Dakota State University, Brookings, South Dakota, United States of America; 5 Department of Pharmacy Practice, College of Pharmacy, South Dakota State University, Brookings, South Dakota, United States of America; University of Santiago de Compostela School of Medicine – CIMUS, Spain

## Abstract

Olanzapine is a first line medication for the treatment of schizophrenia, but it is also one of the atypical antipsychotics carrying the highest risk of weight gain. Metformin was reported to produce significant attenuation of antipsychotic-induced weight gain in patients, while the study of preventing olanzapine-induced weight gain in an animal model is absent. Berberine, an herbal alkaloid, was shown in our previous studies to prevent fat accumulation *in vitro* and *in vivo*. Utilizing a well-replicated rat model of olanzapine-induced weight gain, here we demonstrated that two weeks of metformin or berberine treatment significantly prevented the olanzapine-induced weight gain and white fat accumulation. Neither metformin nor berberine treatment demonstrated a significant inhibition of olanzapine-increased food intake. But interestingly, a significant loss of brown adipose tissue caused by olanzapine treatment was prevented by the addition of metformin or berberine. Our gene expression analysis also demonstrated that the weight gain prevention efficacy of metformin or berberine treatment was associated with changes in the expression of multiple key genes controlling energy expenditure. This study not only demonstrates a significant preventive efficacy of metformin and berberine treatment on olanzapine-induced weight gain in rats, but also suggests a potential mechanism of action for preventing olanzapine-reduced energy expenditure.

## Introduction

Second generation antipsychotics [1] are the first line medications for acute and maintenance therapy of schizophrenia, due to fewer neurological side effects and arguably greater improvement in negative symptoms, when compared with first generation antipsychotics [2], [3]. However, the use of many atypical antipsychotics is well-recognized to be associated with serious metabolic adverse effects, such as weight gain, type II diabetes mellitus, and hyperlipidemia [4]. Olanzapine is a broadly prescribed second generation antipsychotic for the treatment of schizophrenia, bipolar disorder, and depression. However, it is also one of the atypical antipsychotics carrying the highest risk of significant weight gain [5], [6]. A recent review of 34 placebo-controlled clinical studies reported an average weight gain of 3.8–16.2 kg with olanzapine for the duration of three weeks to 12 months [7]. The olanzapine-induced weight gain is accompanied by an increase in human visceral white fat [8], [9]. Besides weight gain and adiposity, olanzapine treatment produces other significant metabolic adverse effects, such as elevated blood glucose and cholesterol levels [10]. The prevalence of metabolic syndrome after second generation antipsychotic treatment was reported to be 20–60%, which is at least double the prevalence rate in the general population [11]–[17]. The significant weight gain and adiposity caused by olanzapine treatment are not only observed in clinical practice, but also well-replicated in a number of rodent studies, including a female Sprague-Dawley (SD) rat model [18], [19].

Management of weight gain and metabolic disturbance caused by atypical antipsychotics includes nutritional, behavioral, and pharmacological interventions. Adjunctive medications are regarded as a critical pharmacologic intervention strategy [20]. Currently, efforts are being put forth in the identification and development of adjunctive medications for the weight gain induced by atypical antipsychotics. A recent review of 32 clinical trials (15 medications, 1482 subjects) reported that only five of the medications significantly ameliorated antipsychotic induced weight gain when compared with placebo treatment. Among those five medications, metformin prevented the greatest weight gain [21]. Although adjunctive metformin treatment has proven to be beneficial in preventing the weight gain resulting from olanzapine treatment in a number of clinical trials [22]–[24], metformin treatment was also shown to prevent olanzapine-induced glucose tolerance in a rat model [25]. Pre-clinical evidence of metformin's efficacy on olanzapine-induced weight gain in an animal study is absent.

Berberine, an isoquinoline alkaloid isolated from multiple herbs such as *Coptis Chinesis* and Goldenseal, is a current over-the-counter (OTC) drug in China for microbial diarrhea treatment. Berberine is also sold in the U.S. market currently as a dietary supplement. Recently, multiple pharmacological effects have been reported to be associated with berberine treatment, including anti-microbial, anti-inflammation, cancer treatment, anti-obesity, anti-diabetic, anti-hyperlipidemia [26], anti-depression[27], and schizophrenia treatment [28]. In the last decade, emerging evidence demonstrated that berberine treatment can induce weight loss in rodents [29], [30] and human subjects [31], [32]. Our previous studies demonstrated that berberine intervention significantly inhibits fat accumulation in cultured adipocytes [33], [34] and diet-induced-obese mice [29]. In a subsequent study utilizing cultured mouse adipocytes, we showed that the berberine treatment completely inhibits fat accumulation induced by the atypical antipsychotics clozapine and risperidone [35].

In this study, we examined the effect and possible mechanism of metformin and berberine treatment on olanzapine-induced weight gain, utilizing a well reported female SD rat model. Rat body weight and white adipose tissue weight were recorded to assess the treatment efficacy. Food intake and the weight of brown adipose tissue were monitored to examine the energy intake and expenditure associated with drug treatment. The expression of genes playing critical roles in energy balance was examined using the *Taq*Man OpenArray gene expression platform.

## Methods

### Animals

Approval from the Institutional Animal Care and Use Committee (IACUC) at South Dakota State University was obtained prior to conducting the animal experiments. Female SD rats (7 weeks old) were purchased from the Charles River Laboratories, housed at 22°C, on a 12-h light-dark cycle, and allowed *ad libitum* access to water and standard laboratory chow diet (Harlan Laboratories 2018 pellets) throughout the study. Following one week of habituation, rats were randomly assigned to four groups where each group was balanced on body weight. We selected 12 rats per group: control group, olanzapine group, olanzapine + berberine group, and olanzapine + metformin group. Rats underwent a one week teaching period to gavage once a day with 0.5% methylcellulose (7.5 ml/kg body weight), followed by drug treatment for two weeks (day 1–14). All rats were housed in the same room. In order to streamline animal necropsy and tissue collection, initial drug treatment was staggered (control and olanzapine + metformin groups firstly, followed by olanzapine and olanzapine + berberine groups one day later). As a result of staggering the initiation of drug treatment, rats were sacrificed in two groups. All rats received drug treatment for the same amount of time. However, the last olanzapine administration was followed by rat sacrifice on the same day and therefore was not a full day of drug treatment. Rat body weight data was collected for thirteen days of drug treatment for all animals.

### Drug treatments

Olanzapine was provided by the Avera Pharmacy at the Avera Behavioral Health Center. The assigned dosage of powdered olanzapine was administered according to a previous report (4 mg/kg once a day for the first week, 8 mg/kg once a day for the second week), which demonstrated a significant weight gain in female SD rats [1]. Berberine treatment at a concentration of 380 mg/kg (once a day) was reported to significantly prevent diet-induced weight gain [36], and that same dosage of berberine was used in this study. To date, there is only one animal study that has tested a co-administration of metformin and olanzapine, which reported that metformin treatment at concentrations of 100 mg/kg or 500 mg/kg significantly prevented olanzapine-induced insulin resistance [37]. In this study, metformin was administered at a concentration of 300 mg/kg. All compounds were suspended in 0.5% methylcellulose and administered by gavage once a day. Food consumption and body weight were recorded daily.

### Tissue collection

After two weeks of drug treatment, rats were anesthetized via CO_2_ anesthesia and exanguinated via cardiac puncture. Cardiac blood samples were collected in EDTA tubes followed by centrifugation to isolate plasma. The plasma samples were stored at −80°C for the subsequent blood glucose and lipid assays. White adipose tissue (perirenal, periovarian, and inguinal fat), interscapular brown adipose tissue, liver, kidney, and spleen were dissected and weighed. The perirenal white adipose, interscapular brown adipose, liver middle lobe, and left thigh skeletal muscle tissues were snap frozen in liquid nitrogen, followed by storage at −80°C for use in subsequent RNA extraction and expression analysis.

### Plasma glucose, triglyceride, and total cholesterol assay

Plasma glucose concentrations were analyzed using the Accutrend Plus system (Roche USA). Triglyceride concentrations were analyzed using the EnzyChrom Triglyceride Assay kit (BioAssay Systems, Hayward, CA, USA). Plasma total cholesterol concentrations were analyzed using the EnzyChrom AF Cholesterol Assay kit BioAssay Systems, Hayward, CA, USA). All experimental assays were completed according to the manufacturer's instructions.

### RNA extraction

Tissue samples (50–100 mg) were suspended in 1 ml Trizol solution (Life Technologies, Carlsbad, CA, USA) and homogenized at room temperature using a tissue homogenizer (Omni International) until no solid tissue was observed. The mRNA was extracted as described in a previous report [29]. RNA was quantified using absorbance at 260 nm with a Nanodrop spectrophotometer (Nanodrop, Wilmington, DE, USA).

### Gene expression analysis

Gene expression analysis was performed with a *Taq*Man OpenArray Real-time RT-PCR System (Life Technologies, Carlsbad, CA, USA). The *Taq*Man OpenArray can be customized to include genes of interest and comes in a wide variety of formats. Prior to the animal experiment, we conducted a thorough literature review and selected 54 candidate genes, plus two endogenous control genes (*β-actin* and *GAPDH*), for the gene expression studies. Any significant expression change in these 54 genes was reported to be associated with olanzapine, metformin, or berberine treatments. The candidate genes selected for this study were shown to be involved in various pathways, including energy/food intake (10 genes), energy expenditure (5 genes), glucose metabolism (7 genes), lipid metabolism (25 genes), inflammation (3 genes), and miscellaneous function (4 genes). A detailed gene list is shown in the Table S1.

The experiment was run in accordance with the manufacturer's protocol. 10 μl RNA (200 ng/μl) was used to synthesize cDNA using the High Capacity cDNA Reverse Transcription Kit (Life technologies, Carlsbad, CA, USA). The cDNA samples and PCR master mix were combined in 384-well plates and loaded onto OpenArray plates using the OpenArray AccuFill Instrument (Life Technologies, Carlsbad, CA, USA). A Real-Time PCR reaction was performed in a sealed OpenArray plate on the BioTrove OpenArray NT Cycler (Life Technologies, Carlsbad, CA, USA). Fluorescence signals were captured and analyzed with the OpenArray Real-Time qPCR Analysis Software Version 1.0.4 (Life Technologies, Carlsbad, CA, USA). Data was imported to DataAssist v3.01 (Life Technologies, Carlsbad, CA, USA) to calculate the relative expression of the selected genes.

### Western Blot analysis

Protein expression of Uncoupling Protein-1 (UCP-1) in brown adipose tissue (BAT) was examined by Western Blot utilizing previously described methods [23]. Protein samples were extracted from BAT homogenized in RIPA buffer (Sigma, Saint Louis, MO, USA) with the addition of Protease Inhibitor Cocktail (Sigma, Saint Louis, MO, USA). Protein concentration was quantified with the Pierce BCA protein assay (Thermo Scientific, Rockford, IL USA). Primary antibodies for human UCP-1 and β-actin, along with the secondary antibodies, were purchased from Santa Cruz Biotechnology (Dallas, TX, USA). Protein was visualized and quantified using the UVP image analysis system (UVP, Upland, CA, USA). The relative protein expression of UCP-1 to β-actin was normalized with the expression level of the control group. Samples from the same treatment group (12 rats per group) were pooled together and then assessed by Western Blot analysis using four replicates.

### Statistical analysis

The data was analyzed by a repeated ANOVA procedure using the Statistical Analysis System (SAS Institute) software. Significant differences between groups were determined using Duncan's multiple range tests at the p<0.05 (*). For OpenArray data analysis, only C_T_ values less than 28 were included in the DataAssist analysis. Our results showed that the expression of *β-actin* was much more stable than *GAPDH* across all rat samples. As a result, the relative quantification of gene expression was normalized to the *β-actin* endogenous control. The Benjamini-Hochberg False Discovery Rate method was used to adjust *p* values in order to correct for the multiple tests.

## Results

### Both metformin and berberine treatment significantly prevented olanzapine-induced weight gain and adiposity

**Figure 1:**
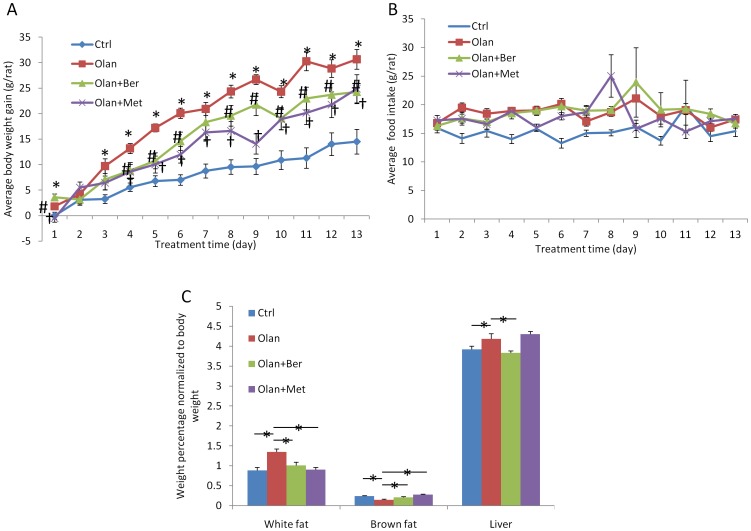
Rat body weight gained during treatment (vehicle, olanzapine, olanzapine + berberine, olanzapine + metformin) (A). * denotes significant difference between olanzapine and control group at P<0.05; # denotes significant difference between olanzapine + berberine and olanzapine group at P<0.05; † denotes significant difference between olanzapine + metformin and olanzapine group at P<0.05. (B) Average food intake (g/rat/day) for each treatment group. (C) Rat white fat, brown fat, and liver weight percentage normalized to body weight in eacg treatment group. * denotes significant difference at P<0.05.

In female Sprague-Dawley (SD) rats, olanzapine treatment induced significant weight gain and adiposity. As shown in Figure 1A and Table 1, olanzapine induced significant weight gain at the end of the drug treatment (14.9% of body weight, 30.65±1.93 grams, P = 0.000015) when compared to vehicle treatment (7.2% of body weight, 14.5±2.41 grams). The amount of total white adipose tissue weight (perirenal fat + periovarian fat+ inguinal fat) as a percentage of after blood draw (ABD) body weight was also significantly increased by olanzapine treatment (52.1%) when compared with vehicle treatment (Figure 1C). The weight increase of subcutaneous white fat and visceral white fat was similar (data not shown). More interestingly, the addition of metformin prevented 35.7% of the body weight gain and 96.3% of the white adipose tissue accumulation induced by the olanzapine treatment. Berberine treatment prevented 39.5% of the body weight gain and 73.9% of the white adipose tissue accumulation when co-administered with olanzapine. At most of the time points during drug treatment, the weight gain attenuation caused by metformin or berberine treatment was very similar. Olanzapine treatment significantly increased the liver weight percentage (6.7%) when normalized to ABD body weight (absolute weight increased from 8.52±0.19 g to 9.54±0.34 g, P = 0.0079). The co-administration of berberine completely prevented the increase in liver weight (8.44±0.21 g, P = 0.0054), while a combination of metformin and olanzapine did not significantly affect liver weight (9.47±0.28 g, P = 0.43) when compared with olanzapine treatment alone. Both kidney and spleen weight were not significantly affected by olanzapine treatment, or olanzapine + metformin treatment, or olanzapine + berberine treatment (data not shown).

**Table 1:** Average weight gain, food intake, white fat weight, brown fat weight, and liver weight in different treatment group (N = 12).

Group	Weight gain (g)	Food intake (g)	White fat (g)	Brown fat (g)	Liver (g)
Ctrl	14.5±2.41	15.24±0.45	1.93±0.17	0.52±0.02	8.52±0.19
Olan	30.65±1.93	18.47±0.39	3.02±0.19	0.33±0.05	9.54±0.34
Olan+Ber	24.26±2.29	18.73±0.53	2.23±0.19	0.46±0.03	8.44±0.21
Olan+Met	24.88±2.74	17.8±0.67	2.0±0.14	0.6±0.03	9.47±0.28

### Both metformin and berberine treatment did not affect food intake but significantly prevented olanzapine-induced brown fat loss

Besides determining that metformin or berberine treatment attenuates olanzapine-induced weight gain and adiposity, we also sought to examine the mechanism of action by monitoring energy input and expenditure. We measured food intake and the weight of interscapular brown adipose tissue (BAT) associated with drug treatment in the female SD rats. Food intake in rats treated with olanzapine was significantly increased compared to vehicle treated rats (see Table 1, 18.47 g/day vs. 15.24 g/day; p = 1.42×10^−5^). However, we did not observe any significant reduction of food intake associated with either metformin or berberine treatment combined with olanzapine treatment, when compared to the olanzapine treatment group (Figure 1B and Table 1). Interestingly, the measurement of BAT weight demonstrated that olanzapine treatment significantly reduced brown fat/body weight percentage by 39.3%. The addition of berberine prevented 70.5% of the BAT weight loss and the addition of metformin completely prevented the BAT weight loss induced by olanzapine (Figure 1C and Table 1).

### Drug treatments did not significantly change blood glucose or lipid levels

As shown in Table 2, drug treatment (Olan only, Olan+Ber, or Olan+Met) did not significantly alter the blood glucose, triglyceride, or total cholesterol levels when compared with the control group.

**Table 2:** Average blood glucose, triglyceride, and total cholesterol levels (mg/dl) in non-fasted female SD rats (N = 12).

Group	Glucose (mg/dl)	Triglyceride (mg/dl)	Total Cholesterol (mg/dl)
Ctrl	120.1±10.8	157.5±7.4	113.0±5.3
Olan	122.8±12.9	152.6±9.9	106.3±7.5
Olan+Ber	117.6±5.4	150.8±11.4	104.6±4.2
Olan+Met	120.8±8.6	149.2±8.8	108.0±10.0

### Gene expression changes associated with drug treatment

We sought to define the molecular mechanism of action for berberine and metformin by examining the gene expression changes associated with drug treatment. Our results demonstrated that food intake was not decreased with the addition of berberine and metformin treatment, indicating that the mechanism of action might be affecting energy expenditure and/or energy storage, rather than altering energy intake. Tissues responsible for energy expenditure or lipid metabolism were selected to run on the OpenArray platform. The tissues selected include brown adipose, skeletal muscle, liver, and perirenal white adipose tissue. Visceral white adipose tissue was selected due to its close association with metabolic diseases compared to subcutaneous white adipose tissue [38].

Among 54 genes investigated on the OpenArray plate, there were 38 genes expressed in brown adipose tissue (Table S2), 41 genes expressed in skeletal muscle tissue (Table S3), 36 genes expressed in liver tissue (Table S4), and 41 genes expressed in white adipose tissue (Table S5). Most of the genes controlling food intake were not expressed in peripheral tissues. As shown in Table S2–S5, the expression of a number of genes was significantly regulated by olanzapine treatment alone or by an addition of berberine/metformin. Those genes mainly function as regulators of energy expenditure, glucose metabolism, and/or lipid metabolism.

We believe the key regulators associated with the preventive effects of berberine/metformin are the genes whose expression was significantly regulated by both the treatment of olanzapine (compared to vehicle treatment) and the treatment of olanzapine + berberine/metformin (compared to olanzapine treatment alone). Those genes (full names and abbreviations) and the relative quantifications of their expression are listed in Table 3. As shown in Table 3, olanzapine treatment alone significantly reduced the expression of three genes controlling energy expenditure (*AMPK* and *UCP3* in brown adipose tissue, and *PGC-1α* in skeletal muscle tissue), while the combination treatment of berberine + olanzapine or metformin + olanzapine significantly increased their expression. The addition of metformin also significantly increased *UCP2* gene expression. In skeletal muscle tissue, the gene expression of *UCP2*, *UCP3*, and *PGC-1α* for the olanzapine + metformin treatment group increased 3.7, 25.2, and 7.9 fold respectively, when compared with the control group. Olanzapine treatment significantly up-regulated the expression of the gluconeogenic gene *PCK1* in liver tissue, while the addition of berberine down-regulated its expression. The expression of *GLUT4* and *PKM2*, two genes playing critical roles in glucose metabolism, was down-regulated by olanzapine treatment, but reversed by the addition of berberine. The treatment, consisting of a combination of metformin and olanzapine, was shown to increase the expression of two genes controlling glucose metabolism (*PCK2* in brown adipose tissue and *GLUT4* in brown adipose, skeletal muscle, and white adipose tissue), while olanzapine treatment alone reduced their expression.

**Table 3:** Relative quantification of gene expression associated with treatment of olanzapine, or olanzapine + berberine, or olanzapine + metformin.

Tissue	Function	Gene	Ctrl	Olan	Olan+Ber	Olan+Met
Brown fat	Energy expenditure	AMP-activated protein kinase-(*AMPK*)	1	0.2984 ***	0.7719 *	1.0536 *
Brown fat	Energy expenditure	Uncoupling protein 3(*UCP3*)	1	0.6258 **	1.0927 *	0.866 *
Brown fat	Glucose metabolism	Glucose transporters 4(*GLUT4*/*SLC2A4*)	1	0.2601 ***	0.5662 **	0.7345 **
Brown fat	Glucose metabolism	Phosphoenolpyruvate carboxykinase 2(*PCK2*)	1	0.5604 *	0.9723	1.1389 *
Brown fat	Glucose metabolism	Pyruvate kinase (*PKM2*)	1	0.3443 ***	0.8652 ***	0.6787
Brown fat	Lipid metabolism	Estrogen sulfotransferase (*EST*/*STE2*)	1	0.2364 ***	0.5898 *	0.2316
Brown fat	Lipid metabolism	Resistin (*RETN*)	1	0.2574 ***	0.3521	0.5312 **
Brown fat	Lipid metabolism	Fatty acid synthase (*FAS*)	1	0.4751 **	0.8386 ***	0.8412 *
Brown fat	Lipid metabolism	Glyerol-3P acyltransferase (*GPAM*)	1	0.4017 ***	0.7997 *	0.8525
Brown fat	Lipid metabolism	Insulin-induced gene 2 (*INSIG2*)	1	0.5394 *	1.2907 *	1.0692 *
Brown fat	Lipid metabolism	Liver X receptor alpha (*LXRA*/*NR1H3*)	1	0.5285 ***	0.867 *	1.1585 *
Brown fat	Lipid metabolism	Acyl-CoA dehydrogenase (*ACADV1*)	1	0.5748 *	0.8586 *	1.1054 *
Brown fat	Lipid metabolism	CCAAT/enhancer binding protein alpha (*C/EBPα*)	1	0.4098 ***	0.6171 *	0.7292 *
Brown fat	Lipid metabolism	Peroxisome proliferator activated receptor gamma(*PPARγ*)	1	0.4375 ***	0.6371	0.6995 *
Liver	Glucose metabolism	Phosphoenolpyruvate carboxykinase 1 (*PCK1*)	1	4.5756 ***	2.4559 *	3.6892
Liver	Lipid metabolism	Insulin-induced gene 2 (*INSIG2*)	1	1.9591 **	1.5637	0.9216 ***
Liver	Lipid metabolism	HMG-CoA reductase (*HMGCR*)	1	0.4599 *	0.8487	0.9876 *
Liver	Lipid metabolism	CCAAT/enhancer binding protein alpha (*C/EBPα*)	1	0.6867 *	1.0102 *	1.0423 **
Skeletal muscle	Energy expenditure	Uncoupling protein 3(*UCP3*)	1	5.2725 **	5.8264	25.2141 *
Skeletal muscle	Energy expenditure	Uncoupling protein 2(*UCP2*)	1	0.4301 *	0.518	3.7121 **
Skeletal muscle	Energy expenditure	PPARy coactivator-1alpha (*PGC-1α*)	1	0.6291 *	1.1504 *	7.93 **
Skeletal muscle	Glucose metabolism	Glucose transporters 4(*GLUT4*/*SLC2A4*)	1	0.6831 **	0.9159 *	5.8091 **
Skeletal muscle	Lipid metabolism	Low-density lipoprotein receptor (*LDLR*)	1	0.4547 **	0.5519	2.5828 **
Skeletal muscle	Lipid metabolism	Sterol regulatory element binding protein-1 (*SREBP-1*)	1	4.1306 **	5.9079	34.5536 **
White fat	Glucose metabolism	Glucose transporters 4(*GLUT4*/*SLC2A4*)	1	0.6919 *	**1.7306**	1.1937 * *
White fat	Lipid metabolism	CCAAT/enhancer binding protein alpha (*C/EBPα*)	1	1.5371 **	2.3521 *	2.7735 *
White fat	Lipid metabolism	Fatty acid synthase (*FAS*)	1	1.5579 **	2.8311 **	2.0223 *
White fat	Lipid metabolism	Glycerol-3P acyltransferase (*GPAM*)	1	1.2941 *	2.9321 **	2.3294 **
White fat	Lipid metabolism	HMG-CoA reductase (*HMGCR*)	1	0.5825 *	0.7815 **	0.7125
White fat	Lipid metabolism	Stearoyl-CoA desaturase (*SCD1*)	1	3.569 **	4.8277 *	3.268
White fat	Lipid metabolism	Sterol regulatory element binding protein-1 (*SREBP-1*)	1	0.5412 *	1.1571 *	1.2275 *
White fat	Lipid metabolism	Apolipoprotein E (*APOE*)	1	0.4891 *	0.4128 *	0.6056 *

Bold numbers are significant expression changes (Olan vs. Ctrl, or Olan+Ber vs. Olan, or Olan+Met vs. Olan). * denotes significant expression change at P<0.05; ** denotes significant expression change at P<0.01; ***denotes significant expression change at P<0.001.

The expression of a number of genes involved in lipid metabolism was also significantly regulated by the treatment of olanzapine. Specifically, the expression of a key lipogenic gene, the sterol regulatory element binding protein-1 gene (*SREBP-1*), was down-regulated by olanzapine treatment (Table 3). The addition of berberine or metformin to olanazapine treated rats significantly up-regulated *SREBP-1* gene expression. The combination treatment, either with berberine or metformin, restored the *SREBP-1* expression to normal levels (control group).

### Olanzapine treatment significantly reduced UCP-1 protein expression while the addition of berberine or metformin restored its expression to control levels

**Figure 2:**
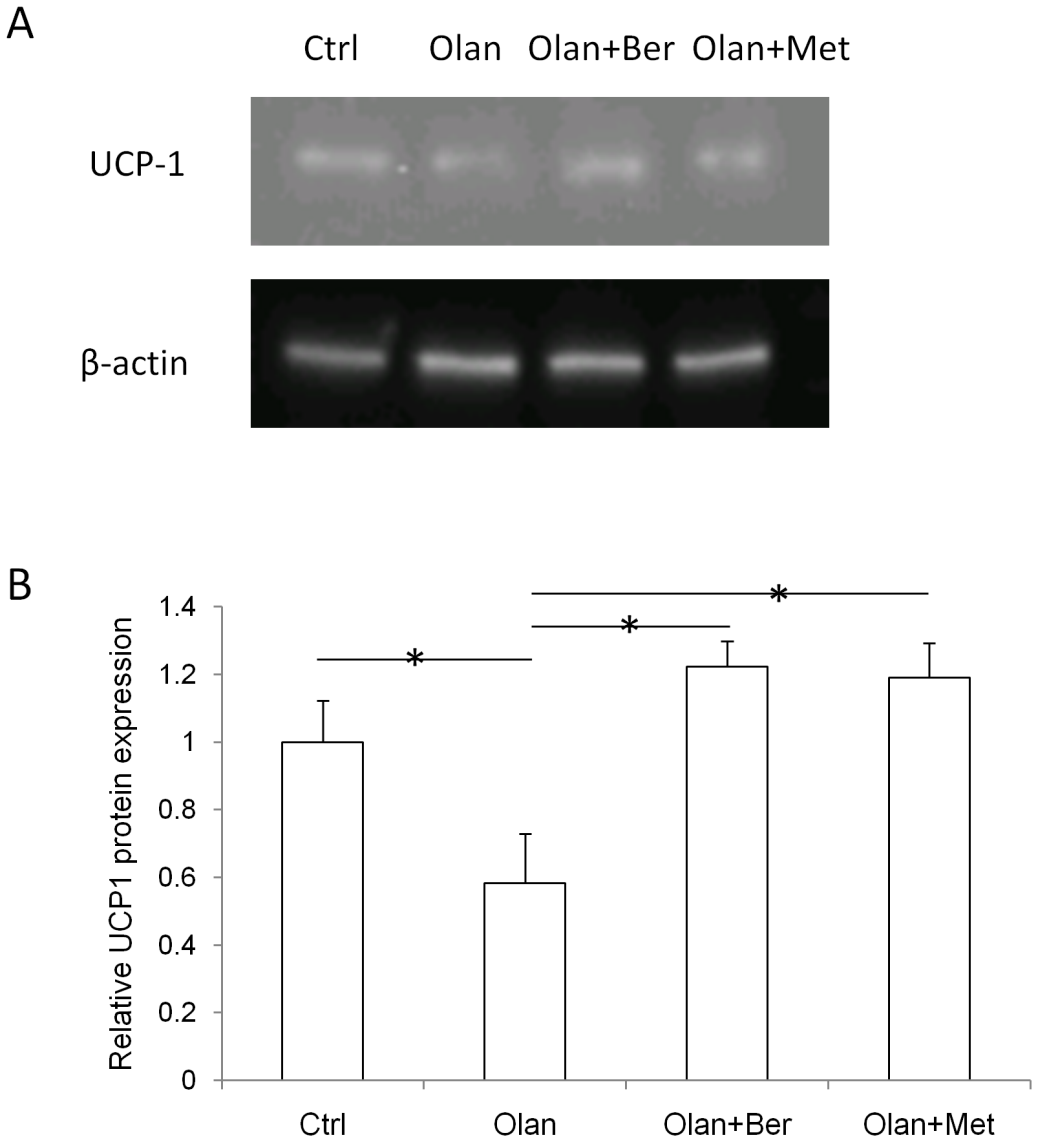
UCP-1 and β-actin protein expression in each treatment group (vehicle, olanzapine, olanzapine + berberine, olanzapine + metformin) (A). Quantification of relative protein expression of UCP-1 to β-actin, when normalized with the control group (B). * denotes significant difference at P<0.05.

As shown in Figure 2, olanzapine treatment significantly decreased UCP-1 protein expression (by 41.7%), when compared with the vehicle treatment (control group). The combination treatment (either Olan+Ber or Olan+Met) significantly increased UCP-1 protein expression when compared with olanzapine treatment. The addition of either berberine or metformin restored the olanzapine-reduced UCP-1 protein expression to normal (control group).

## Discussion

Antipsychotic-induced metabolic adverse effects are not only risk factors for cardiovascular disease and insulin resistance leading to increased morbidity and mortality, but also, these effects impair the patient's adherence to treatment [39]. Many efforts have been ongoing for the identification and development of adjunctive medications to prevent the weight gain induced by antipsychotics including olanzapine. Metformin is a first line medication to treat type II diabetes, with a putative mechanism of inhibitory hepatic gluconeogenesis [40]. Interestingly, a number of recent clinical trials reported a significant attenuation efficacy of metformin treatment on olanzapine-induced weight gain, with an unindentified mechanism of action [21]. We are the first group to observe the clinical preventive efficacy of metformin treatment in an olanzapine-induced weight gain rat model. This finding in rats also enabled us to further investigate the mechanism of metformin's action. Berberine treatment was reported to reduce fat accumulation *in vitro*, *in vivo*, and in human subjects [32], [36]. In our rat study, the results demonstrated that berberine treatment prevented 39.5% of body weight gain and 73.9% of white adipose tissue accumulation induced by olanzapine, which is a comparable efficacy to metformin treatment (weight gain and tissue weights were normalized with rat body weight). We are the first group to demonstrate that a dietary supplement, berberine, significantly prevents olanzapine-induced weight gain *in vivo*, which might imply a potential effectiveness on human subjects. So far multiple *in vivo* studies have reported excellent effectiveness of berberine treatment on non-alcoholic fatty liver disease [41], [42]. Our data also showed that the addition of berberine completely prevented the significant increase (by 6.7%) of liver weight induced by olanzapine, suggesting a possible benefit of berberine treatment on liver function disturbed by the olanzapine treatment.

We sought to investigate the mechanism of action for metformin and berberine from the perspective of energy balance. The increased energy storage induced by olanzapine was extensively considered a result of both increased energy intake (such as hyperphagia) [43], [44] and reduced energy expenditure (such as decreased thermogenesis) [45], [46]. Our results showed that olanzapine treatment increased food intake by 21.2%, enhanced fat accumulation by 52.1%, and reduced brown fat amount by 39.3%, when compared with the control group. Neither a co-administration of metformin nor berberine significantly prevented the increased food intake induced by olanzapine. Metformin treatment has been reported to reduce food intake in rodent studies [47]. Our results showed that the combined treatment of metformin and olanzapine did not significantly prevent increased food intake induced by olanazpine, which was probably due to the specific drug dosages used in our study. Although some studies reported a reduced food intake associated with berberine treatment in rodents [48], other articles showed that berberine treatment did not affect food intake [36], [49]. Our results showed that berberine + olanzapine treatment did not prevent olanzapine-increased food intake.

Interestingly, the addition of metformin prevented 96.3% of the white adipose accumulation induced by olanzapine, which is associated with a complete restoration of brown fat loss caused by olanzapine. The addition of berberine prevented 73.9% of the white adipose accumulation with a 70.5% attenuation of brown fat loss induced by olanzapine. This is the first evidence that adjunctive berberine or metformin treatment significantly prevents olanzapine-induced brown fat weight loss. The weight of rodent brown fat is shown to be associated with changes in core body temperature [50]. Our tissue weight data strongly suggest the mechanism of action for metformin or berberine occurs by increasing energy expenditure or directly reducing energy storage, rather than inhibition of energy intake. On the other hand, since the treatment of metformin or berberine did not moderate olanzapine-induced food intake, it is not surprising that the drug treatment only partially prevented olanzapine-induced weight gain.

With the aim of elucidating the molecular mechanism of metformin and berberine's efficacy, we performed gene expression assays utilizing the tissues responsible for energy expenditure and storage. Brown adipose tissue is the key tissue responsible for thermogenesis (heat production) in rodents. Among five genes controlling thermogenesis in brown adipose tissue (BAT), olanzapine treatment resulted in significantly reduced expression of three genes (*AMPK*, *UCP2*, and *UCP3*). Reduced expression of *AMPK*, *UCP2*, and *UCP3* suggests thermogenesis would be decreased, which is consistent with olanzapine-reduced BAT temperature reported by another research group [51]. Olanzapine treatment did not significantly change the mRNA expression of a key thermogenic effector, *UCP1*, in brown adipose tissue. Just recently it was reported that there is a low correlation between levels of *UCP1* mRNA and UCP1 protein [52], therefore we decided to perform a Western Blot assay to assess UCP1 protein expression. The results demonstrated that the regulation of UCP1 protein expression associated with drug treatment was similar to the expression changes of other thermogenic genes. Interestingly, the addition of metformin or berberine to olanzapine treatment significantly reversed the effect of olanzapine on the expression of two of the three genes (*AMPK* and *UCP3*). Co-administration of metformin also significantly increased *UCP1* gene expression when compared with olanzapine treatment alone. Our BAT gene expression results suggest that the co-administration of metformin or berberine might prevent olanzapine-reduced thermogenesis, which is consistent with our finding of BAT weight changes associated with the drug treatments. In another critical energy expenditure tissue, skeletal muscle, olanzapine treatment reduced the expression of *PGC-1α* and *UCP2* but increased the expression of *UCP3*, thus the overall effect of olanzapine treatment on energy expenditure in skeletal muscle tissue is not clear. Interestingly, the addition of metformin to the olanzapine treatment increased the expression of all five genes, while the addition of berberine increased the expression of *PGC-1α* in skeletal muscle tissue, when compared with olanzapine treatment alone. Based on the observed gene expression data, the co-administration of metformin or berberine would increase energy expenditure in skeletal muscle tissue. Although the BAT weight and gene expression data strongly suggest increasing energy expenditure for berberine or metformin's action, a limitation of this study is the absence of a direct measurement of energy expenditure.

Olanzapine treatment showed an increase in blood glucose levels in human subjects [53] as well as in rodents [54]. Both metformin and berberine are potent anti-diabetic compounds. A combination of metformin and olanzapine was reported to decrease insulin resistance in an animal study [37], while the effects of berberine combined with olanzapine on glucose levels currently remains unexamined *in vivo*. Our gene expression analysis showed that the expression of *PCK1*, a key gene controlling hepatic gluconeogenesis, significantly increased due to the olanzapine treatment. Conversely, the addition of berberine significantly reduced the expression. Glucose transporter 4 (*GLUT4*) facilitates cellular glucose intake. The expression of *GLUT4* in brown adipose tissue, skeletal muscle tissue, and white adipose tissue was significantly reduced by olanzapine treatment, while co-administration of metformin or berberine significantly increased its expression in the three tissues. Our gene expression data suggested a beneficial effect of metformin or berberine on olanzapine-increased glucose levels. Increased fasting blood glucose and lipid levels are associated with olanzapine treatment in human subjects [55] and in animal studies [56]. The female SD rat model we utilized is the most broadly reported animal model for olanzapine-induced weight gain, however this model is not ideal for olanzapine-induced hyperglycemia or hyperlipidemia. Many articles reported no change in glucose/lipid levels [57] or made no reference to glucose/lipids data at all [19], [58] in the olanzapine-induced weight gain animal models. We measured the glucose and lipid levels and did not find significant changes.

Olanzapine-induced adiposity is associated with expression changes in a number of lipogenic and/or adipogenic genes [59]. Those genes are included in our OpenArray expression platform data. Gene expression was analyzed within different rat tissue samples. In BAT, our gene expression data showed that olanzapine treatment significantly decreased the expression of ten adipogenic or lipogenic genes, without up-regulation of genes involved in lipid metabolism. This is consistent with a recent report from another research group stating that second generation antipsychotics inhibited the expression of a number of adipogenic and lipogenic genes [60]. Our results also showed that the addition of either metformin or berberine restored the expression of eight of these ten genes to near normal levels. How olanzapine-decreased expression of lipogenic genes in BAT contributes to the olanzapine-induced BAT weight loss is not clear, though the addition of berberine or metformin bringing those genes' expression to control group levels might be beneficial. Both metformin and berberine are reported to be *AMPK* activators [58], [61]. In this study, we did not examine the activity of *AMPK*, but we did observe that olanzapine-reduced *AMPK* expression in BAT was restored close to control group levels, when co-administrated with metformin or berberine. In peripheral white adipose tissue, increased AMPK activity was shown to be associated with weight loss and reduced lipogenic gene expression [62]. Interestingly, decreased AMPK activity in the rat hypothalamus was reported to be associated with weight loss as well [63]. How the AMPK activity (including gene expression) in BAT affects body weight and lipogenic gene expression is not clear. Future studies will be needed to elucidate this.

In white adipose tissue, olanzapine treatment was shown to significantly increase the expression of *C/EBPα*, *FAS*, and *GPAM*. This suggests increased adipogenesis and lipogenesis. Interestingly, our results also showed that olanzapine treatment reduced the expression of a key lipogenic transcription factor, *SREBP-1*, to 0.54 fold in white adipose tissue. A recent article from another research group reported that acute olanzapine administration to rats resulted in rapid up-regulation of *SREBP-1* in white adipose tissue, followed by a late phase down-regulation (after 3 hours of treatment) [64]. Our rats were sacrificed on average after 4 hours of drug treatment, which could be a reason for the rebound effect of *SREBP-1* expression. Our previous study [35] showed that berberine prevented fat accumulation induced by atypical antipsychotics (clozapine and risperidone) *in vitro*, associated with the down-regulation of lipogenic *SREBP-1* gene expression. Interestingly, in this animal study, our data showed that the attenuation of olanzapine-induced weight gain by the addition of berberine was associated with increased *SREBP-1* expression, when compared with olanzapine treatment alone. The expression of *SREBP-1* in white adipose tissue was initially increased by clozapine or olanzapine treatment, followed by down-regulation [64]. Our animal study showed a down-regulated expression of *SREBP-1* by olanzapine treatment, while the addition of berberine treatment restored its expression close to normal levels (control group). Although the exact reason for the discrepancy in *SREBP-1* gene expression between *in vitro* and *in vivo* studies is not clear, it seemed that berberine treatment in both studies reversed the effects of antipsychotics on *SREBP-1* expression, which is associated with a prevention of antipsychotic-induced fat accumulation.

In skeletal muscle tissue it is very interesting to note that the expression of SREBP-1 was increased 4.1 fold by olanzapine treatment, while the addition of metformin increased its expression 34.6 fold. Although little is known about the function of SREBP-1 in skeletal muscle, a few recent studies suggest that over-expression of *SREBP-1* induces skeletal muscle atrophy [65], [66]. Attention should be paid to the possible muscle loss side effect caused by olanzapine or metformin treatments based on this expression finding.

In conclusion, both metformin and berberine treatments demonstrated a significant prevention of olanzapine-induced weight gain and adiposity in SD female rats. Olanzapine treatment significantly reduced rat BAT weight and down-regulated the expression of key thermogenic genes including *AMPK* and *UCP3*. The addition of metformin or berberine significantly prevented BAT weight reduction and up-regulated *AMPK* and *UCP3* expression. Our findings on the regulation of BAT weight and regulation of gene expression indicate a potential mechanism of stimulating energy expenditure through metformin or berberine treatment.

## Supporting Information

Table S1 **Genes designated on OpenArray plate.**
(PDF)

Table S2 **Relative quantification (RQ) of gene expression in rat brown adipose tissue.**
(PDF)

Table S3 **Relative quantification (RQ) of gene expression in rat skeletal muscle tissue.**
(PDF)

Table S4 **Relative quantification (RQ) of gene expression in rat liver tissue.**
(PDF)

Table S5 **Relative quantification (RQ) of gene expression in rat white adipose tissue.**
(PDF)

## References

[1] Albaugh VL, Henry CR, Bello NT, Hajnal A, Lynch SL, et al (2006) Hormonal and metabolic effects of olanzapine and clozapine related to body weight in rodents. Obesity (Silver Spring) 14: 36–51. 16493121 10.1038/oby.2006.6 PMC2761763

[2] Miyamoto S, Duncan GE, Marx CE, Lieberman JA (2005) Treatments for schizophrenia: a critical review of pharmacology and mechanisms of action of antipsychotic drugs. Mol Psychiatry 10: 79–104. 15289815 10.1038/sj.mp.4001556

[3] Jarskog LF, Miyamoto S, Lieberman JA (2007) Schizophrenia: new pathological insights and therapies. Annu Rev Med 58: 49–61. 16903799 10.1146/annurev.med.58.060904.084114

[4] Newcomer JW (2004) Metabolic risk during antipsychotic treatment. Clin Ther 26: 1936–1946. 15823759 10.1016/j.clinthera.2004.12.003

[5] Muench J, Hamer AM (2010) Adverse effects of antipsychotic medications. Am Fam Physician 81: 617–622. 20187598

[6] Citrome L, Holt RI, Walker DJ, Hoffmann VP (2011) Weight gain and changes in metabolic variables following olanzapine treatment in schizophrenia and bipolar disorder. Clin Drug Investig 31: 455–482. 10.2165/11589060-000000000-00000 21495734

[7] De Hert M, Detraux J, van Winkel R, Yu W, Correll CU (2012) Metabolic and cardiovascular adverse effects associated with antipsychotic drugs. Nat Rev Endocrinol 8: 114–126. 10.1038/nrendo.2011.156 22009159

[8] Blouin M, Tremblay A, Jalbert ME, Venables H, Bouchard RH, et al (2008) Adiposity and eating behaviors in patients under second generation antipsychotics. Obesity (Silver Spring) 16: 1780–1787. 18535555 10.1038/oby.2008.277

[9] Gilles M, Hentschel F, Paslakis G, Glahn V, Lederbogen F, et al (2010) Visceral and subcutaneous fat in patients treated with olanzapine: a case series. Clin Neuropharmacol 33: 248–249. 20838214 10.1097/WNF.0b013e3181f0ec33

[10] Lindenmayer JP, Czobor P, Volavka J, Citrome L, Sheitman B, et al (2003) Changes in glucose and cholesterol levels in patients with schizophrenia treated with typical or atypical antipsychotics. Am J Psychiatry 160: 290–296. 12562575 10.1176/appi.ajp.160.2.290

[11] De Hert MA, van Winkel R, Van Eyck D, Hanssens L, Wampers M, et al (2006) Prevalence of the metabolic syndrome in patients with schizophrenia treated with antipsychotic medication. Schizophr Res 83: 87–93. 16481149 10.1016/j.schres.2005.12.855

[12] Haupt DW (2006) Differential metabolic effects of antipsychotic treatments. Eur Neuropsychopharmacol 16 Suppl 3 S149–155. 16872808 10.1016/j.euroneuro.2006.06.003

[13] Newcomer JW, Haupt DW (2006) The metabolic effects of antipsychotic medications. Can J Psychiatry 51: 480–491. 16933585 10.1177/070674370605100803

[14] Saddichha S, Manjunatha N, Ameen S, Akhtar S (2008) Metabolic syndrome in first episode schizophrenia - a randomized double-blind controlled, short-term prospective study. Schizophr Res 101: 266–272. 18262771 10.1016/j.schres.2008.01.004

[15] Shirzadi AA, Ghaemi SN (2006) Side effects of atypical antipsychotics: extrapyramidal symptoms and the metabolic syndrome. Harv Rev Psychiatry 14: 152–164. 16787887 10.1080/10673220600748486

[16] Thakore JH (2004) Metabolic disturbance in first-episode schizophrenia. Br J Psychiatry Suppl 47: S76–79. 10.1192/bjp.184.47.s76 15056598

[17] Toalson P, Ahmed S, Hardy T, Kabinoff G (2004) The Metabolic Syndrome in Patients With Severe Mental Illnesses. Prim Care Companion J Clin Psychiatry 6: 152–158. 15361918 10.4088/pcc.v06n0402 PMC514841

[18] Goudie AJ, Smith JA, Halford JC (2002) Characterization of olanzapine-induced weight gain in rats. J Psychopharmacol 16: 291–296. 12503827 10.1177/026988110201600402

[19] Liebig M, Gossel M, Pratt J, Black M, Haschke G, et al (2010) Profiling of energy metabolism in olanzapine-induced weight gain in rats and its prevention by the CB1-antagonist AVE1625. Obesity (Silver Spring) 18: 1952–1958. 20168311 10.1038/oby.2010.17

[20] Faulkner G, Cohn TA (2006) Pharmacologic and nonpharmacologic strategies for weight gain and metabolic disturbance in patients treated with antipsychotic medications. Can J Psychiatry 51: 502–511. 16933587 10.1177/070674370605100805

[21] Maayan L, Vakhrusheva J, Correll CU (2010) Effectiveness of medications used to attenuate antipsychotic-related weight gain and metabolic abnormalities: a systematic review and meta-analysis. Neuropsychopharmacology 35: 1520–1530. 20336059 10.1038/npp.2010.21 PMC3055458

[22] Praharaj SK, Jana AK, Goyal N, Sinha VK (2011) Metformin for olanzapine-induced weight gain: a systematic review and meta-analysis. Br J Clin Pharmacol 71: 377–382. 21284696 10.1111/j.1365-2125.2010.03783.x PMC3045546

[23] Wu RR, Zhao JP, Guo XF, He YQ, Fang MS, et al (2008) Metformin addition attenuates olanzapine-induced weight gain in drug-naive first-episode schizophrenia patients: a double-blind, placebo-controlled study. Am J Psychiatry 165: 352–358. 18245179 10.1176/appi.ajp.2007.07010079

[24] Chen CH, Chiu CC, Huang MC, Wu TH, Liu HC, et al (2008) Metformin for metabolic dysregulation in schizophrenic patients treated with olanzapine. Prog Neuropsychopharmacol Biol Psychiatry 32: 925–931. 18082302 10.1016/j.pnpbp.2007.11.013

[25] Boyda HN, Procyshyn RM, Asiri Y, Wu C, Wang CK, et al (2013) Antidiabetic-drug combination treatment for glucose intolerance in adult female rats treated acutely with olanzapine. Prog Neuropsychopharmacol Biol Psychiatry 48C: 170–176. 10.1016/j.pnpbp.2013.10.006 24140931

[26] Vuddanda PR, Chakraborty S, Singh S (2010) Berberine: a potential phytochemical with multispectrum therapeutic activities. Expert Opin Investig Drugs 19: 1297–1307. 10.1517/13543784.2010.517745 20836620

[27] Hu Y, Ehli EA, Hudziak JJ, Davies GE (2012) Berberine and evodiamine influence serotonin transporter (5-HTT) expression via the 5-HTT-linked polymorphic region. Pharmacogenomics J 12: 372–378. 21647174 10.1038/tpj.2011.24

[28] Kulkarni SK, Dhir A (2010) Berberine: a plant alkaloid with therapeutic potential for central nervous system disorders. Phytother Res 24: 317–324. 19998323 10.1002/ptr.2968

[29] Hu Y, Davies GE (2010) Berberine inhibits adipogenesis in high-fat diet-induced obesity mice. Fitoterapia 81: 358–366. 19861153 10.1016/j.fitote.2009.10.010

[30] Zhang X, Zhao Y, Zhang M, Pang X, Xu J, et al (2012) Structural changes of gut microbiota during berberine-mediated prevention of obesity and insulin resistance in high-fat diet-fed rats. PLoS One 7: e42529. 22880019 10.1371/journal.pone.0042529 PMC3411811

[31] Zhang Y, Li X, Zou D, Liu W, Yang J, et al (2008) Treatment of type 2 diabetes and dyslipidemia with the natural plant alkaloid berberine. J Clin Endocrinol Metab 93: 2559–2565. 18397984 10.1210/jc.2007-2404

[32] Hu Y, Ehli EA, Kittelsrud J, Ronan PJ, Munger K, et al (2012) Lipid-lowering effect of berberine in human subjects and rats. Phytomedicine 19: 861–867. 22739410 10.1016/j.phymed.2012.05.009

[33] Hu Y, Davies GE (2009) Berberine increases expression of GATA-2 and GATA-3 during inhibition of adipocyte differentiation. Phytomedicine 16: 864–873. 19403287 10.1016/j.phymed.2009.03.002

[34] Hu Y, Fahmy H, Zjawiony JK, Davies GE (2010) Inhibitory effect and transcriptional impact of berberine and evodiamine on human white preadipocyte differentiation. Fitoterapia 81: 259–268. 19799972 10.1016/j.fitote.2009.09.012

[35] Hu Y, Kutscher E, Davies GE (2010) Berberine inhibits SREBP-1-related clozapine and risperidone induced adipogenesis in 3T3-L1 cells. Phytother Res 24: 1831–1838. 20564506 10.1002/ptr.3204

[36] Lee YS, Kim WS, Kim KH, Yoon MJ, Cho HJ, et al (2006) Berberine, a natural plant product, activates AMP-activated protein kinase with beneficial metabolic effects in diabetic and insulin-resistant states. Diabetes 55: 2256–2264. 16873688 10.2337/db06-0006

[37] Boyda HN, Procyshyn RM, Tse L, Hawkes E, Jin CH, et al (2012) Differential effects of 3 classes of antidiabetic drugs on olanzapine-induced glucose dysregulation and insulin resistance in female rats. J Psychiatry Neurosci 37: 407–415. 22640703 10.1503/jpn.110140 PMC3493097

[38] Wajchenberg BL (2000) Subcutaneous and visceral adipose tissue: their relation to the metabolic syndrome. Endocr Rev 21: 697–738. 11133069 10.1210/edrv.21.6.0415

[39] Tschoner A, Engl J, Laimer M, Kaser S, Rettenbacher M, et al (2007) Metabolic side effects of antipsychotic medication. Int J Clin Pract 61: 1356–1370. 17627711 10.1111/j.1742-1241.2007.01416.x

[40] Viollet B, Guigas B, Sanz Garcia N, Leclerc J, Foretz M, et al (2012) Cellular and molecular mechanisms of metformin: an overview. Clin Sci (Lond) 122: 253–270. 22117616 10.1042/CS20110386 PMC3398862

[41] Xing LJ, Zhang L, Liu T, Hua YQ, Zheng PY, et al (2011) Berberine reducing insulin resistance by up-regulating IRS-2 mRNA expression in nonalcoholic fatty liver disease (NAFLD) rat liver. Eur J Pharmacol 668: 467–471. 21839075 10.1016/j.ejphar.2011.07.036

[42] Yang QH, Hu SP, Zhang YP, Xie WN, Li N, et al (2011) Effect of berberine on expressions of uncoupling protein-2 mRNA and protein in hepatic tissue of non-alcoholic fatty liver disease in rats. Chin J Integr Med 17: 205–211. 21359922 10.1007/s11655-011-0668-4

[43] Kluge M, Schuld A, Himmerich H, Dalal M, Schacht A, et al (2007) Clozapine and olanzapine are associated with food craving and binge eating: results from a randomized double-blind study. J Clin Psychopharmacol 27: 662–666. 18004133 10.1097/jcp.0b013e31815a8872

[44] Case M, Treuer T, Karagianis J, Hoffmann VP (2010) The potential role of appetite in predicting weight changes during treatment with olanzapine. BMC Psychiatry 10: 72. 20840778 10.1186/1471-244X-10-72 PMC2945973

[45] Kreuzer P, Landgrebe M, Wittmann M, Hajak G, Schecklmann M, et al (2012) [Hypothermia under olanzapine treatment: clinical case series and review of current literature]. Nervenarzt 83: 630–637. 21626387 10.1007/s00115-011-3310-y

[46] Kudoh A, Takase H, Takazawa T (2004) Chronic treatment with antipsychotics enhances intraoperative core hypothermia. Anesth Analg 98: : 111–115, table of contents. 10.1213/01.ANE.0000093313.16711.5E 14693598

[47] D'Agostino G, La Rana G, Russo R, Sasso O, Iacono A, et al (2007) Acute intracerebroventricular administration of palmitoylethanolamide, an endogenous peroxisome proliferator-activated receptor-alpha agonist, modulates carrageenan-induced paw edema in mice. J Pharmacol Exp Ther 322: 1137–1143. 17565008 10.1124/jpet.107.123265

[48] Wang Y, Campbell T, Perry B, Beaurepaire C, Qin L (2011) Hypoglycemic and insulin-sensitizing effects of berberine in high-fat diet- and streptozotocin-induced diabetic rats. Metabolism 60: 298–305. 20304443 10.1016/j.metabol.2010.02.005

[49] Zhang M, Lv X, Li J, Meng Z, Wang Q, et al (2012) Sodium caprate augments the hypoglycemic effect of berberine via AMPK in inhibiting hepatic gluconeogenesis. Mol Cell Endocrinol 363: 122–130. 22922125 10.1016/j.mce.2012.08.006 PMC3795615

[50] Makino T, Kato K, Mizukami H (2009) Processed aconite root prevents cold-stress-induced hypothermia and immuno-suppression in mice. Biol Pharm Bull 32: 1741–1748. 19801837 10.1248/bpb.32.1741

[51] Stefanidis A, Verty AN, Allen AM, Owens NC, Cowley MA, et al (2009) The role of thermogenesis in antipsychotic drug-induced weight gain. Obesity (Silver Spring) 17: 16–24. 19107124 10.1038/oby.2008.468

[52] Nedergaard J, Cannon B (2013) UCP1 mRNA does not produce heat. Biochim Biophys Acta 1831: 943–949. 23353596 10.1016/j.bbalip.2013.01.009

[53] Schreiner A, Niehaus D, Shuriquie NA, Aadamsoo K, Korcsog P, et al (2012) Metabolic effects of paliperidone extended release versus oral olanzapine in patients with schizophrenia: a prospective, randomized, controlled trial. J Clin Psychopharmacol 32: 449–457. 22722501 10.1097/JCP.0b013e31825cccad

[54] Smith GC, Vickers MH, Shepherd PR (2011) Olanzapine effects on body composition, food preference, glucose metabolism and insulin sensitivity in the rat. Arch Physiol Biochem 117: 241–249. 21671852 10.3109/13813455.2011.576681

[55] Feng S, Melkersson K (2012) Metabolic parameters and long-term antipsychotic treatment: a comparison between patients treated with clozapine or olanzapine. Neuro Endocrinol Lett 33: 493–498. 23090266

[56] Ikegami M, Ikeda H, Ishikawa Y, Ohsawa M, Ohashi T, et al.. (2013) Olanzapine induces glucose intolerance through the activation of AMPK in the mouse hypothalamus. Eur J Pharmacol. 10.1016/j.ejphar.2013.08.006 23973646

[57] Fell MJ, Anjum N, Dickinson K, Marshall KM, Peltola LM, et al (2007) The distinct effects of subchronic antipsychotic drug treatment on macronutrient selection, body weight, adiposity, and metabolism in female rats. Psychopharmacology (Berl) 194: 221–231. 17581744 10.1007/s00213-007-0833-9

[58] Sejima E, Yamauchi A, Nishioku T, Koga M, Nakagama K, et al (2011) A role for hypothalamic AMP-activated protein kinase in the mediation of hyperphagia and weight gain induced by chronic treatment with olanzapine in female rats. Cell Mol Neurobiol 31: 985–989. 21681559 10.1007/s10571-011-9663-8 PMC11498405

[59] Skrede S, Ferno J, Vazquez MJ, Fjaer S, Pavlin T, et al (2012) Olanzapine, but not aripiprazole, weight-independently elevates serum triglycerides and activates lipogenic gene expression in female rats. Int J Neuropsychopharmacol 15: 163–179. 21854679 10.1017/S1461145711001271

[60] Oh JE, Cho YM, Kwak SN, Kim JH, Lee KW, et al (2012) Inhibition of mouse brown adipocyte differentiation by second-generation antipsychotics. Exp Mol Med 44: 545–553. 22809901 10.3858/emm.2012.44.9.062 PMC3465748

[61] Russo GL, Russo M, Ungaro P (2013) AMP-activated protein kinase: a target for old drugs against diabetes and cancer. Biochem Pharmacol 86: 339–350. 23747347 10.1016/j.bcp.2013.05.023

[62] Lee MS, Kim IH, Kim CT, Kim Y (2011) Reduction of body weight by dietary garlic is associated with an increase in uncoupling protein mRNA expression and activation of AMP-activated protein kinase in diet-induced obese mice. J Nutr. 141: 1947–53. 10.3945/jn.111.146050 21918057

[63] Martínez de Morentin PB, Whittle AJ, Fernø J, Nogueiras R, Diéguez C, et al (2012) Nicotine induces negative energy balance through hypothalamic AMP-activated protein kinase. Diabetes 61: 807–17. 22315316 10.2337/db11-1079 PMC3314364

[64] Jassim G, Skrede S, Vazquez MJ, Wergedal H, Vik-Mo AO, et al (2012) Acute effects of orexigenic antipsychotic drugs on lipid and carbohydrate metabolism in rat. Psychopharmacology (Berl) 219: 783–794. 21748251 10.1007/s00213-011-2397-y PMC3259403

[65] Dessalle K, Euthine V, Chanon S, Delarichaudy J, Fujii I, et al (2012) SREBP-1 transcription factors regulate skeletal muscle cell size by controlling protein synthesis through myogenic regulatory factors. PLoS One 7: e50878. 23226416 10.1371/journal.pone.0050878 PMC3511457

[66] Lecomte V, Meugnier E, Euthine V, Durand C, Freyssenet D, et al (2010) A new role for sterol regulatory element binding protein 1 transcription factors in the regulation of muscle mass and muscle cell differentiation. Mol Cell Biol 30: 1182–1198. 20028734 10.1128/MCB.00690-09 PMC2820883

