# Supplementary material for: Metformin and Berberine Prevent Olanzapine-Induced Weight Gain in Rats
Source: PLoS One. 2014 Mar 25;9(3):e93310. doi: 10.1371/journal.pone.0093310 (PMC3965561; doi:10.1371/journal.pone.0093310)
Supplement: Table S1 — Genes designated on OpenArray plate. (PDF) [file pone.0093310.s001.pdf]

Table S1: Genes designated on OpenArray plate

Function	Gene name	ABI inventory #
Energy intake	Neuropeptide Y (NPY)	Rn01410145_m1
	Gouti related protein (AgRP)	Rn01431703_g1
	Proopiomelanocortin (POMC)	Rn00595020_m1
	Melanin-concentrating hormone receptor (MCHR1)	Rn00755896_m1
	Hypothalamic orexin neuropeptide (Hcrt)	Rn00565995_m1
	Serotonin receptor 2A (HTR2A)	Rn00568473_m1
	Serotonin receptor 2C (HTR2C)	Rn00562748_m1
	Serotoin transporter (SERT)	Rn00564737_m1
	Brain-derived neurotrophin factor (BDNF)	Rn02531967_s1
	Histamine H1 receptor (Hrh1)	Rn00566691_s1
Energy expenditure	AMP-activated protein kinase-(AMPK)	Rn00576935_m1
	Uncoupling protein 1(UCP1)	Rn00562126_m1
	Uncoupling protein 2(UCP2)	Rn01754856_m1
	Uncoupling protein 3(UCP3)	Rn00565874_m1
	PPARy coactivator-1alpha (PGC-1α)	Rn00580241_m1
Glucose metabolism	Phosphoenolpyruvate carboxykinase 1 (Pck1)	Rn01529014_m1
	Phosphoenolpyruvate carboxykinase 2(Pck2)	Rn03648110_m1
	11 beta-hydroxysteroid dehydrogenase type 1 (Hsd11b1)	Rn00567167_m1
	Glucose transporters 4(GLUT4/Slc2a4)	Rn00562597_m1
	Pyruvate kinase (Pkm2)	Rn00583975_m1
	Glycogen phosphorylase (Pygl)	Rn00573974_m1
	Insulinotropic polypeptide (Gip)	Rn00571500_m1
Lipid metabolism: adipogenesis	Peroxisome proliferator activated receptor gamma(PPARγ)	Rn00440945_m1
	CCAAT/enhancer binding protein alpha (C/EBPα)	Rn00560963_s1
	GATA binding protein 2 (GATA2)	Rn00583735_m1
	GATA binding protein 3 (GATA3)	Rn00484683_m1
Lipid metabolism: lipogenesis	Sterol regulatory element binding protein-1 (SREBP-1)	Rn01495769_m1
	Fatty acid synthase (FAS)	Rn00569117_m1
	Insulin-induced gene 2 (INSIG2)	Rn00710111_m1
	Low-density lipoprotein receptor (LDLR)	Rn00598442_m1
	Citrate transporter/carrier (Slc13a5)	Rn00596734_m1
	Acetyl-co-A carboxylase alpha (Acaca)	Rn00573474_m1
	Acetyl-co-A carboxylase belta (Acacb)	Rn00588290_m1
	Glycerol-3P acyltransferase (GPAM)	Rn00568620_m1
	Stearoyl-CoA desaturase (SCD1)	Rn00594894_g1
	HMG-CoA reductase (Hmgcr)	Rn00565598_m1
Lipid metabolism: lipolysis	Peroxisome proliferator activated receptor alpha(PPARα)	Rn00566193_m1
	Liver X receptor alpha (LXRa/Nr1h3)	Rn00581185_m1
	Hormone-sensitive lipase (HSL/Lipe)	Rn00563444_m1
	Apolipoprotein E (ApoE)	Rn00593680_m1
	Acyl-CoA dehydrogenase (Acadvl)	Rn00563649_m1
	Acyl-CoA oxidase (Acox1)	Rn01460628_m1
	Insulin Receptor (IssR)	Rn01637243_m1
	Phospholipase C, beta 1 (PLCB1)	Rn01514511_m1
Lipid metabolism: adipokine	Adiponectin (Adipoq)	Rn00595250_m1
	Leptin (Lep)	Rn00565158_m1
	Resistin (Retn)	Rn00595224_m1
Inflammation	Tumor necrosis factor α (TNFα/Tnf)	Rn00562055_m1
	Interleukin 6 (IL-6)	Rn01410330_m1
	C-Reactive Protein (CRP)	Rn00567307_g1
Others	Estrogen sulfotransferase (EST/ste2)	Rn01510633_m1
	Mitogen-activated protein kinase 14 (MAPK14)	Rn00578842_m1
	MAPK8 (c-jun N-terminal)	Rn01453358_m1
	Mitogen-activated protein kinase 1 (MAPK1)	Rn00587719_m1
Endogenous control	Beta-actin (Actb)	Rn00667869_m1
	GAPDH (Gapdh)	Rn01775763_g1
