# Supplementary material for: Metformin and Berberine Prevent Olanzapine-Induced Weight Gain in Rats
Source: PLoS One. 2014 Mar 25;9(3):e93310. doi: 10.1371/journal.pone.0093310 (PMC3965561; doi:10.1371/journal.pone.0093310)
Supplement: Table S2 — Relative quantification (RQ) of gene expression in rat brown adipose tissue. (PDF) [file pone.0093310.s002.pdf]

Table S2: Relative quantification (RQ) of gene expression in rat brown adipose tissue

Function	Gene	RQ (Ctrl)	RQ (Olan)	RQ (Olan+Ber)	RQ (Olan+Met)
Energy expenditure	Uncoupling protein 1(UCP1)	1	0.8203	0.9344	**1.2592**
	Uncoupling protein 3(UCP3)	1	**0.6258**	**1.0927**	**0.866**
	AMP-activated protein kinase-(AMPK)	1	**0.2984**	**0.7719**	**1.0536**
	PPARy coactivator-1alpha (PGC-1α)	1	1.1581	1.1025	1.1912
	Uncoupling protein 2(UCP2)	1	**0.5695**	0.8397	0.6633
Energy intake	Neuropeptide Y (NPY)	1	2.395	2.4346	2.8334
Glucose metabolism	Glucose transporters 4(GLUT4/Slc2a4)	1	**0.2601**	**0.5662**	**0.7345**
	Glycogen phosphorylase (Pygl)	1	**0.3439**	0.5908	0.5154
	Pyruvate kinase (Pkm2)	1	**0.3443**	**0.8652**	0.6787
	Phosphoenolpyruvate carboxykinase 1 (Pck1)	1	0.9306	0.8284	0.906
	Phosphoenolpyruvate carboxykinase 2(Pck2)	1	**0.5604**	0.9723	**1.1389**
Lipid metabolism	Peroxisome proliferator activated receptor gamma(PPARγ)	1	**0.4375**	0.6371	**0.6995**
	GATA binding protein 3 (GATA3)	1	1.6323	0.9087	1.13
	CCAAT/enhancer binding protein alpha (C/EBPα)	1	**0.4098**	**0.6171**	**0.7292**
	Leptin (Lep)	1	**0.3359**	0.3252	0.4196
	Resistin (Retn)	1	**0.2574**	0.3521	**0.5312**
	Adiponectin (Adipoq)	1	0.8291	0.8006	0.9466
	HMG-CoA reductase (Hmgcr)	1	0.8969	1.5273	1.2317
	Glycerol-3P acyltransferase (GPAM)	1	**0.4017**	**0.7997**	0.8525
	Fatty acid synthase (FAS)	1	**0.4751**	**0.8386**	**0.8412**
	Acetyl-co-A carboxylase alpha (Acaca)	1	**0.264**	0.6341	0.0962
	Acetyl-co-A carboxylase belta (Acacb)	1	0.8683	1.1859	1.1465
	Stearoyl-CoA desaturase (SCD1)	1	0.8177	**1.6264**	**2.0457**
	Low-density lipoprotein receptor (LDLR)	1	**0.4447**	0.9503	0.4462
	Insulin-induced gene 2 (INSIG2)	1	**0.5394**	**1.2907**	**1.0692**
	Sterol regulatory element binding protein-1 (SREBP-1)	1	0.2831	**0.5465**	0.4502
	Acyl-CoA dehydrogenase (Acadvl)	1	**0.5748**	**0.8586**	**1.1054**
	Peroxisome proliferator activated receptor alpha(PPARα)	1	0.9472	0.9398	**1.342**
	Liver X receptor alpha (LXRa/Nr1h3)	1	**0.5285**	**0.867**	**1.1585**
	Apolipoprotein E (ApoE)	1	1.1833	1.5608	0.6811
	Acyl-CoA oxidase (Acox1)	1	0.75	1.3394	1.3902
	Insulin Receptor (IssR)	1	0.4399	0.6096	0.4864
Others	Mitogen-activated protein kinase 14 (MAPK14)	1	**0.5219**	**0.7842**	0.7379
	Mitogen-activated protein kinase 1 (MAPK1)	1	**0.3312**	0.6639	0.4108
	MAPK8 (c-jun N-terminal)	1	0.835	0.9767	**1.1969**
	Estrogen sulfotransferase (EST/ste2)	1	**0.2364**	**0.5898**	0.2316

Bold numbers are significant at $P<0.05$ when compared between Olan vs. Ctrl group, or Olan+Ber vs. Olan group, or Olan+Met vs. Olan group
