# Supplementary material for: Metformin and Berberine Prevent Olanzapine-Induced Weight Gain in Rats
Source: PLoS One. 2014 Mar 25;9(3):e93310. doi: 10.1371/journal.pone.0093310 (PMC3965561; doi:10.1371/journal.pone.0093310)
Supplement: Table S3 — Relative quantification (RQ) of gene expression in rat skeletal muscle tissue. (PDF) [file pone.0093310.s003.pdf]

Table S3: Relative quantification (RQ) of gene expression in rat skeletal muscle tissue

Function	Gene	RQ (Ctrl)	RQ (Olan)	RQ (Olan+Ber)	RQ (Olan+Met)
Energy expenditure	Uncoupling protein 3(UCP3)	1	**5.2725**	5.8264	**25.2141**
	AMP-activated protein kinase-(AMPK)	1	1.1366	1.4445	**10.6677**
	PPARγ coactivator-1alpha (PGC-1α)	1	**0.6291**	**1.1504**	**7.93**
	Uncoupling protein 2(UCP2)	1	**0.4301**	0.518	**3.7121**
Energy intake	Melanin-concentrating hormone receptor (MCHR1)	1	0.7247	1.1298	**5.9765**
	Neuropeptide Y (NPY)	1	1.683	2.1522	10.9093
	Brain-derived neurotrophin factor (BDNF)	1	0.6225	1.4633	**13.6618**
Glucose metabolism	Glucose transporters 4(GLUT4/Slc2a4)	1	**0.6831**	**0.9159**	**5.8091**
	11 beta-hydroxysteroid dehydrogenase type 1 (Hsd11b1)	1	0.7911	0.9622	**9.0137**
	Glycogen phosphorylase (Pygl)	1	0.6689	0.9799	5.6517
	Pyruvate kinase (Pkm2)	1	0.7276	0.9569	**5.4085**
	Phosphoenolpyruvate carboxykinase 1 (Pck1)	1	1.737	1.4742	12.7736
	Phosphoenolpyruvate carboxykinase 2(Pck2)	1	1.5627	1.4059	**7.6118**
Lipid metabolism	Peroxisome proliferator activated receptor gamma(PPARγ)	1	1.4398	1.4256	**7.8838**
	GATA binding protein 3 (GATA3)	1	12.4083	1.5022	7.8392
	CCAAT/enhancer binding protein alpha (C/EBPα)	1	0.9374	1.3561	**18.6854**
	GATA binding protein 2 (GATA2)	1	0.9611	1.149	**6.1346**
	Resistin (Retn)	1	0.9184	1.7295	8.2144
	Adiponectin (Adipoq)	1	1.5436	1.4222	8.8381
	HMG-CoA reductase (Hmgcr)	1	0.7524	0.8707	**5.3612**
	Glycerol-3P acyltransferase (GPAM)	1	1.1697	1.4581	**8.8462**
	Fatty acid synthase (FAS)	1	3.6696	1.5659	7.3338
	Acetyl-co-A carboxylase alpha (Acaca)	1	1.7466	2.0467	13.0438
	Acetyl-co-A carboxylase belta (Acacb)	1	0.9371	**1.2831**	**8.4324**
	Stearoyl-CoA desaturase (SCD1)	1	2.4175	2.9121	13.6912
	Low-density lipoprotein receptor (LDLR)	1	**0.4547**	0.5519	**2.5828**
	Insulin-induced gene 2 (INSIG2)	1	0.8104	1.0371	**7.7335**
	Sterol regulatory element binding protein-1 (SREBP-1)	1	**4.1306**	5.9079	**34.5536**
	Acyl-CoA dehydrogenase (Acadvl)	1	0.9049	1.1229	**6.6496**
	Peroxisome proliferator activated receptor alpha(PPARα)	1	0.7399	1.0053	**7.2291**
	Liver X receptor alpha (LXRa/Nr1h3)	1	0.9602	1.1923	**7.9329**
	Apolipoprotein E (ApoE)	1	1.5352	1.2249	**8.2145**
	Acyl-CoA oxidase (Acox1)	1	0.8647	**1.1199**	**6.6402**
	Phospholipase C, beta 1 (PLCB1)	1	1.1448	0.9957	no data
	Insulin Receptor (IssR)	1	0.9254	0.989	**7.9966**
Others	Mitogen-activated protein kinase 14 (MAPK14)	1	0.7673	**1.0373**	**6.669**
	Mitogen-activated protein kinase 1 (MAPK1)	1	0.7964	1.0565	**4.5759**
	MAPK8 (c-jun N-terminal)	1	0.9182	1.057	**6.4065**
	Estrogen sulfotransferase (EST/ste2)	1	0.9817	1.2528	**9.1726**

Bold numbers are significant at $P<0.05$ when compared between Olan vs. Ctrl group, or Olan+Ber vs. Olan group, or Olan+Met vs. Olan group
