# Supplementary material for: Metformin and Berberine Prevent Olanzapine-Induced Weight Gain in Rats
Source: PLoS One. 2014 Mar 25;9(3):e93310. doi: 10.1371/journal.pone.0093310 (PMC3965561; doi:10.1371/journal.pone.0093310)
Supplement: Table S4 — Relative quantification (RQ) of gene expression in rat liver tissue. (PDF) [file pone.0093310.s004.pdf]

Table S4: Relative quantification (RQ) of gene expression in rat liver tissue

Function	Gene	RQ (Ctrl)	RQ (Olan)	RQ (Olan+Ber)	RQ (Olan+Met)
Energy expenditure	AMP-activated protein kinase-(AMPK)	1	0.9725	1.1593	1.0984
	PPARγ coactivator-1alpha (PGC-1α)	1	**1.6593**	2.531	2.7791
	Uncoupling protein 2(UCP2)	1	0.9871	0.9787	1.0092
Energy intake	Neuropeptide Y (NPY)	1	0.4508	0.4051	0.497
Glucose metabolism	11 beta-hydroxysteroid dehydrogenase type 1 (Hsd11b1)	1	1.0782	1.4803	0.5996
	Glycogen phosphorylase (Pygl)	1	0.9947	1.2045	1.0406
	Phosphoenolpyruvate carboxykinase 1 (Pck1)	1	**4.5756**	**2.4559**	3.6892
	Phosphoenolpyruvate carboxykinase 2(Pck2)	1	**1.5112**	1.4758	1.5623
Inflammation	C-Reactive Protein (CRP)	1	**1.2941**	1.4143	1.2061
Lipid metabolism	Peroxisome proliferator activated receptor gamma(PPARγ)	1	0.7454	0.7925	0.9284
	GATA binding protein 3 (GATA3)	1	1.1369	1.1734	1.2346
	CCAAT/enhancer binding protein alpha (C/EBPα)	1	**0.6867**	**1.0102**	**1.0423**
	GATA binding protein 2 (GATA2)	1	1.3388	1.309	1.1717
	HMG-CoA reductase (Hmgcr)	1	**0.4599**	0.8487	**0.9876**
	Glycerol-3P acyltransferase (GPAM)	1	0.999	0.9361	0.7248
	Fatty acid synthase (FAS)	1	**0.4548**	0.6634	0.7528
	Acetyl-co-A carboxylase alpha (Acaca)	1	0.9848	1.4051	1.7197
	Acetyl-co-A carboxylase belta (Acacb)	1	0.9064	1.1723	0.9437
	Stearoyl-CoA desaturase (SCD1)	1	0.3991	0.6632	0.8532
	Citrate transporter/carrier (Slc13a5)	1	1.3694	1.4929	1.1423
	Low-density lipoprotein receptor (LDLR)	1	0.8952	1.0698	1.1579
	Insulin-induced gene 2 (INSIG2)	1	**1.9591**	1.5637	**0.9216**
	Sterol regulatory element binding protein-1 (SREBP-1)	1	0.8302	**1.4149**	1.3042
	Acyl-CoA dehydrogenase (Acadvl)	1	**1.2746**	1.357	1.3059
	Peroxisome proliferator activated receptor alpha(PPARα)	1	1.3311	1.4724	1.2987
	Liver X receptor alpha (LXRa/Nr1h3)	1	1.1069	1.1864	1.1911
	Apolipoprotein E (ApoE)	1	1.1971	1.3812	1.2497
	Acyl-CoA oxidase (Acox1)	1	**1.4725**	1.5201	1.3848
	Phospholipase C, beta 1 (PLCB1)	1	1.1252	1.251	**0.1716**
	Insulin Receptor (IssR)	1	0.8849	1.0633	1.017
Others	Mitogen-activated protein kinase 14 (MAPK14)	1	0.8256	**1.1154**	1.0912
	Mitogen-activated protein kinase 1 (MAPK1)	1	1.1105	1.1865	1.1235
	MAPK8 (c-jun N-terminal)	1	**1.4227**	1.7085	1.3491
	Estrogen sulfotransferase (EST/ste2)	1	0.9062	1.0904	1.0763

Bold numbers are significant at $P<0.05$ when compared between Olan vs. Ctrl group, or Olan+Ber vs. Olan group, or Olan+Met vs. Olan group
