# Supplementary material for: Metformin and Berberine Prevent Olanzapine-Induced Weight Gain in Rats
Source: PLoS One. 2014 Mar 25;9(3):e93310. doi: 10.1371/journal.pone.0093310 (PMC3965561; doi:10.1371/journal.pone.0093310)
Supplement: Table S5 — Relative quantification (RQ) of gene expression in rat white adipose tissue. (PDF) [file pone.0093310.s005.pdf]

Table S5: Relative quantification (RQ) of gene expression in rat white adipose tissue

Function	Gene	RQ (Ctrl)	RQ (Olan)	RQ (Olan+Ber)	RQ (Olan+Met)
Energy expenditure	Uncoupling protein 1(UCP1)	1	1.1257	0.9187	**0.5976**
	Uncoupling protein 3(UCP3)	1	0.9076	0.8517	0.774
	AMP-activated protein kinase-(AMPK)	1	0.7135	0.7842	0.9662
	PPARy coactivator-1alpha (PPGC-1α)	1	1.0719	0.9314	1.1531
	Uncoupling protein 2(UCP2)	1	**0.4168**	0.4534	0.5022
Energy intake	Serotoin transporter (SERT)	1	0.4689	0.4384	1.0554
	Neuropeptide Y (NPY)	1	0.9503	0.7849	**1.9328**
Glucose metabolism	Glucose transporters 4(GLUT4/Slc2a4)	1	**0.6919**	**1.7306**	**1.1937**
	11 beta-hydroxysteroid dehydrogenase type 1 (Hsd11b1)	1	1.3417	1.6528	1.6998
	Glycogen phosphorylase (Pygl)	1	1.0645	**1.6001**	**1.6395**
	Pyruvate kinase (Pkm2)	1	0.8344	**1.157**	0.7264
	Phosphoenolpyruvate carboxykinase 1 (Pck1)	1	**1.9056**	1.7782	2.0638
	Phosphoenolpyruvate carboxykinase 2(Pck2)	1	0.7283	1.001	**1.7455**
Lipid metabolism	Peroxisome proliferator activated receptor gamma(PPARγ)	1	1.2541	**1.9716**	**2.0387**
	GATA binding protein 3 (GATA3)	1	1.3531	**0.5908**	**0.3241**
	CCAAT/enhancer binding protein alpha (C/EBPα)	1	**1.5371**	**2.3521**	**2.7735**
	GATA binding protein 2 (GATA2)	1	0.9927	0.6027	**1.2382**
	Leptin (Lep)	1	1.4334	**3.1486**	**3.1338**
	Resistin (Retn)	1	1.0035	**1.6825**	1.4622
	Adiponectin (Adipoq)	1	**1.9209**	2.1533	2.6437
	HMG-CoA reductase (Hmgcr)	1	**0.5825**	**0.7815**	0.7125
	Glycerol-3P acyltransferase (GPAM)	1	**1.2941**	**2.9321**	**2.3294**
	Fatty acid synthase (FAS)	1	**1.5579**	**2.8311**	**2.0223**
	Acetyl-co-A carboxylase alpha (Acaca)	1	0.7424	1.7355	**1.3982**
	Acetyl-co-A carboxylase belta (Acacb)	1	**1.327**	1.7079	1.1045
	Stearoyl-CoA desaturase (SCD1)	1	**3.569**	**4.8277**	3.268
	Low-density lipoprotein receptor (LDLR)	1	**0.48**	0.5398	0.4104
	Insulin-induced gene 2 (INSIG2)	1	0.4742	1.1118	**1.224**
	Sterol regulatory element binding protein-1 (SREBP-1)	1	**0.5412**	**1.1571**	**1.2275**
	Acyl-CoA dehydrogenase (Acadvl)	1	0.8832	**1.0685**	0.9995
	Peroxisome proliferator activated receptor alpha(PPARα)	1	1.0601	0.8645	0.9966
	Liver X receptor alpha (LXRa/Nr1h3)	1	1.5782	1.8477	2.0035
	Apolipoprotein E (ApoE)	1	**0.4891**	**0.4128**	**0.6056**
	Acyl-CoA oxidase (Acox1)	1	1.0737	**1.3532**	1.3132
	Phospholipase C, beta 1 (PLCB1)	1	**0.3736**	0.1912	
	Insulin Receptor (IssR)	1	0.7932	**1.2356**	1.4171
Others	Mitogen-activated protein kinase 14 (MAPK14)	1	**0.5432**	0.6093	0.7106
	Mitogen-activated protein kinase 1 (MAPK1)	1	**0.6574**	0.6445	0.7301
	MAPK8 (c-jun N-terminal)	1	0.9281	0.9632	0.961
	Estrogen sulfotransferase (EST/ste2)	1	0.9996	1.5356	**1.6695**

Bold numbers are significant at $P<0.05$ when compared between Olan vs. Ctrl group, or Olan+Ber vs. Olan group, or Olan+Met vs. Olan group
